# Prevalence of benzodiazepines prescription in the dental office across the Eastern region of Saudi Arabia

**DOI:** 10.12688/f1000research.165185.1

**Published:** 2025-07-25

**Authors:** Mohammed E. Alabbad, Ashwin C. Shetty, Subraya G. Bhat, Faisal E. Aljofi, Khalid Almas

**Affiliations:** 1 Resident, Fellowship in Periodontics, College of Dentistry, Imam Abdulrahman Bin Faisal University, Dammam, Eastern Province, Saudi Arabia; 2 Department of Dental Education, Imam Abdulrahman Bin Faisal University, Dammam, Eastern Province, Saudi Arabia; 3 Department of Preventive Dental Sciences, College of Dentistry, Imam Abdulrahman Bin Faisal University, Dammam, Eastern Province, Saudi Arabia

**Keywords:** Prevalence; Oral sedatives; Dental offices; Drug prescription; Saudi Arabia

## Abstract

**Background:**

Anesthesia and sedation services for dental outpatients are commonly indicated for the management of fear, anxiety, and phobia, usually with children and patients with some medical conditions. To date, there has been no information on the prevalence of oral sedatives (Benzodiazepines, OBZ) used for dental patients in the Eastern region of Saudi Arabia. Hence, this study aimed to determine the prevalence of oral sedatives used for dental patients in the Eastern region of the Kingdom of Saudi Arabia.

**Material and Methods:**

An electronic survey (though Google forms) was sent to the practitioners in the Eastern region of Saudi Arabia via e-mail/social media. The calculated representative sample size required for the study was 327. All participants were registered with the Saudi Commission for Health Specialties (SCFHS). The questionnaire consisted of 28 questions, which included demographics and oral benzodiazepine prescription patterns. Data obtained were analyzed using the SPSS (Version 23.0) software.

**Results:**

The completed response was received from 378 dentists who participated in the study. We found that 6.9% (n=26) had prescribed oral benzodiazepines to adult patients for anxiety management (as oral pre-medication or conscious sedation). Specialty dentists/trainees, private sector dentists, and non-Kingdom of Saudi Arabia (KSA), foreign trained/qualified dentists had prescribed more OBZs. A statistically significant association was found between barriers to prescribing for managing anxious patients and the type of dentist (p<.05).

**Conclusion:**

This cross-sectional study concluded that the prevalence of prescribing OBZ in the Eastern region of Saudi Arabia is low. The most important barriers identified were the lack of training and formal qualifications.

## 1. Introduction

Anesthesia and sedation services for dental outpatients are commonly indicated for the management of fear, anxiety, and phobia. Additional specific indications for sedation include patients with cognitive impairment who are unable to cooperate, young or emotionally challenged children who cannot cooperate, patients with motor dysfunction (for example, uncontrollable gagging), and extensive surgical procedures or other situations in which local anesthesia may provide insufficient pain control. Data presented to date revealed numerous areas of consensus. Foremost among these was the general agreement that there is a strong need and demand for oral sedation services in dentistry that are not always met by available resources. ^1^

A study in Saudi Arabia reported a dental anxiety prevalence of 51.6% among dental patients, while the mean anxiety score of the patients was 11.39. ^2^ Another study from China reported a 74% prevalence of dental fear among 1203 patients, 27.3% of the total had periodontal disease. The average score of Corach’s Dental Anxiety Scales and Dental Fear Survey for patients with periodontal disease was significantly higher than those without periodontal disease. ^3^ A similar finding was reported by a study from Nepal with a 95.49% prevalence of anxiety among patients visiting for periodontal therapy. ^4^ Furthermore, periodontal patients treated with non-surgical therapy had higher anxiety, depression, and stress, and poorer well-being than surgical therapy. However, surgical patients reported a higher level of pain during the second week, and greater consumption of analgesics during the second and fourth weeks. ^5^ A study that evaluated the impact of anxiety on pain perception reported that the level of presurgical anxiety affects pain perception for both periodontal and implant surgery. ^6^

One of the methods employed for the management of fear and anxiety is the use of oral benzodiazepines. A summary table of the most common benzodiazepines used in dentistry is presented in Table 1. The prevalence of prescribing Oral Benzodiazepines (OBZ) has reached 10,640 prescriptions by the year 2016 in Australia. ^7^ In general, benzodiazepine prescriptions were higher in the United States of America compared to England and Australia. Furthermore, diazepam was the most commonly prescribed oral benzodiazepine in these three countries. ^8^ A recent study in Tanzania reported that the practice of sedation in dentistry is very low due to a lack of knowledge. ^9^ The Saudi Food and Drug Administration (FDA) considers OBZ a controlled drug, which includes any drug or chemical regulated by the authority for its potential for abuse, addiction, or misuse. ^8^ To date, there has been no information on the prevalence of oral sedatives used for dental patients in Saudi Arabia (Eastern region). Hence, this study aimed to determine the prevalence of oral sedatives used for dental patients in the Eastern region of Saudi Arabia.

**Table 1.** Common oral benzodiazepines.

Drug	Dosage			Onset	Duration
Diazepam	2.0 mg	5.0 mg	10 mg	Fast	Long
Triazolam	0.25 mg	-	-	Fast	Short
Alprazolam	0.25 mg	0.50 mg	1.0 mg	Intermediate	Intermediate
Lorazepam	0.50 mg	1.0 mg	2.0 mg	Intermediate	Intermediate
Oxazepam	10 mg	15 mg	30 mg	Slow	Short

## 2. Materials and Methods

### 2.1 Study design

This is a cross-sectional study, where an electronic survey was sent to the practitioners in the Eastern region of Saudi Arabia via e-mail/social media.

### 2.2 Data collection

A validated English language questionnaire from a previous study was adapted for data collection. ^10, 16^ The questionnaire consisted of 28 questions, which included demographics and oral benzodiazepine prescription patterns. The written permission was obtained from the developer of the questionnaire to use it via email. No identifying information was included in the questionnaire. The questionnaire consisted of both open-ended and closed questions. The questionnaire was piloted on 5% of the total sample to check the length, clarity, and ambiguity of the questions. These responses were excluded from the study. The purpose of the study was clearly explained to the participants, and the consent was obtained from participants before participating in the study.

### 2.3 Inclusion and exclusion criteria

• Only the dental practitioners registered with the SCFHS were included.
• Incomplete questionnaires were discarded.

### 2.4 Sample size calculation

The sample data for the study were extracted from the last updated yearly statistical book from the Ministry of Health. According to the available data from the registry, the number of practitioners in the Eastern region was 3277, to which we added 800 additional practitioners to compensate for the recent years’ inclusion and the number of practitioners in other governmental sectors. Assuming proportion of sedative prescriptions by dentists = 0.36, total registered dentists in Eastern region (N = 4000), on a 5% margin of error, 95% confidence interval, and 80% power, the representative sample size is 327.

### 2.5 Ethical considerations

This study was approved by the Institutional Review Board (IRB) at Imam Abdulrahman Bin Faisal University with the letter number **IRB-PGS-2024-02-250** on 23 April, 2024.

### 2.6 Statistical analysis

Data obtained were analyzed using the SPSS (Version 23.0, Chicago, Illinois, USA) software. The descriptive data were expressed in terms of means with standard deviations and percentages. Chi-square and Fisher’s Exact tests were employed to assess the association between the variables. Statistical significance was set at p ≤ 0.05.

## 3. Results

In total, 378 eligible dentists participated in the study, of which 6.9% (n = 26) had prescribed oral benzodiazepines (temazepam or diazepam) to adult patients for anxiety management (as oral pre-medication or conscious sedation). The results indicate the total number of respondents who answered the related question ( Table 2).

**Table 2.** Demographics of survey respondents.

Demographic		Total number of respondents (n = 378)	Proportion who had prescribed OBZs (95% CI)
Sex	Male	286	7 (4.3-10.6)
	Female	92	6.5 (2.4-13.7)
Type of dentist	General dentist	206	4.9 (2.4-8.8)
	Specialty dentist/trainee	172	9.3 (5.4-14.7)
Workplace	Public sector	314	6.4 (3.9-9.7)
	Private sector	64	9.4 (3.5-19.3)
Country qualified	KSA	324	6.2 (3.8-9.4)
	Non-KSA	54	11.1 (4.2-22.6)
Year qualified	1985–1994	10	*
	1995–2004	32	18.8 (7.2-36.4)
	2005–2014	72	8.3 (3.1-17.3)
	2015–2024	262	5.3 (3.0-8.8)

* =Numbers too small for statistical analysis.

**Table 3.** Demographics and association between barriers to prescribing for managing anxious patients.

	Gender n (%)		Type of dentist n (%)		Work place n (%)		Year qualified n (%)	
	Male	Female	General dentist	Specialty dentist/trainee	Public sector	Private sector	1985-2004	2005-2024
**Inadequate training**	126 (47.4)	36 (43.9)	100 (51.5)	62 (40.3)	130 (44.5)	32 (57.1)	6 (16.7)	156 (50.3)
**No formal sedation qualification**	108 (40.6)	36 (43.9)	86 (44.3)	58 (37.7)	122 (41.8)	22 (39.3)	10 (27.8)	132 (42.6)
**Prefer other anxiety management approaches**	74 (27.8)	26 (31.7)	40 (20.6)	60 (39.0)	82 (28.1)	18 (32.1)	14 (38.9)	86 (27.7)
**Medicolegal risk**	40 (15.0)	16 (19.5)	28 (14.4)	28 (18.2)	48 (16.4)	8 (14.3)	8 (22.2)	48 (15.5)
**Lack of confidence**	42 (15.8)	6 (7.3)	28 (14.4)	20 (13.0)	40 (13.7)	8 (14.3)	2 (5.6)	46 (14.8)
**Other (please comment)**	30 (11.3)	12 (14.6)	18 (9.3)	24 (15.6)	34 (11.6)	8 (14.3)	6 (16.7)	36 (11.6)
**Do not believe they are effective**	14 (5.3)	2 (2.4)	11 (5.7)	5 (3.2)	15 (5.1)	1 (1.8)	6 (16.7)	10 (3.2)
**Feel they are unsafe to use**	10 (3.8)	4 (4.9)	6 (3.1)	8 (5.2)	12 (4.1)	2 (3.6)	4 (11.1)	8 (2.6)
**Not adequately remunerated**	12 (4.5)	2 (2.4)	8 (4.1)	6 (3.9)	12 (4.1)	2 (3.6)	2 (5.6)	12 (3.9)
**p-value **	.488		.002		.811		*	

* =Numbers too small for statistical analysis.

### 3.1 Demographics

Most respondents were men (n = 286, 75.7%), aged 22–40 years (n = 318, 84.1%). General dental practitioners (n = 206, 54.5%), working in the public sector (n = 314, 83.1%), had qualified in KSA (n = 324, 85.7%), and qualified between the years 2015-2024 (n = 262, 69.3%).

A very small difference existed in OBZ prescribing experience between males and females (7% vs 6.5%) (see Table 2). Specialty dentists/trainees, private sector dentists, and non-KSA qualified dentists were more likely to have prescribed OBZs. Those qualified before 2004 were three times more likely to have prescribed OBZs than those qualified since 2015 (18.8% compared with 5.3%).

### 3.2 Patterns of OBZ prescribing

**Figure 1.**
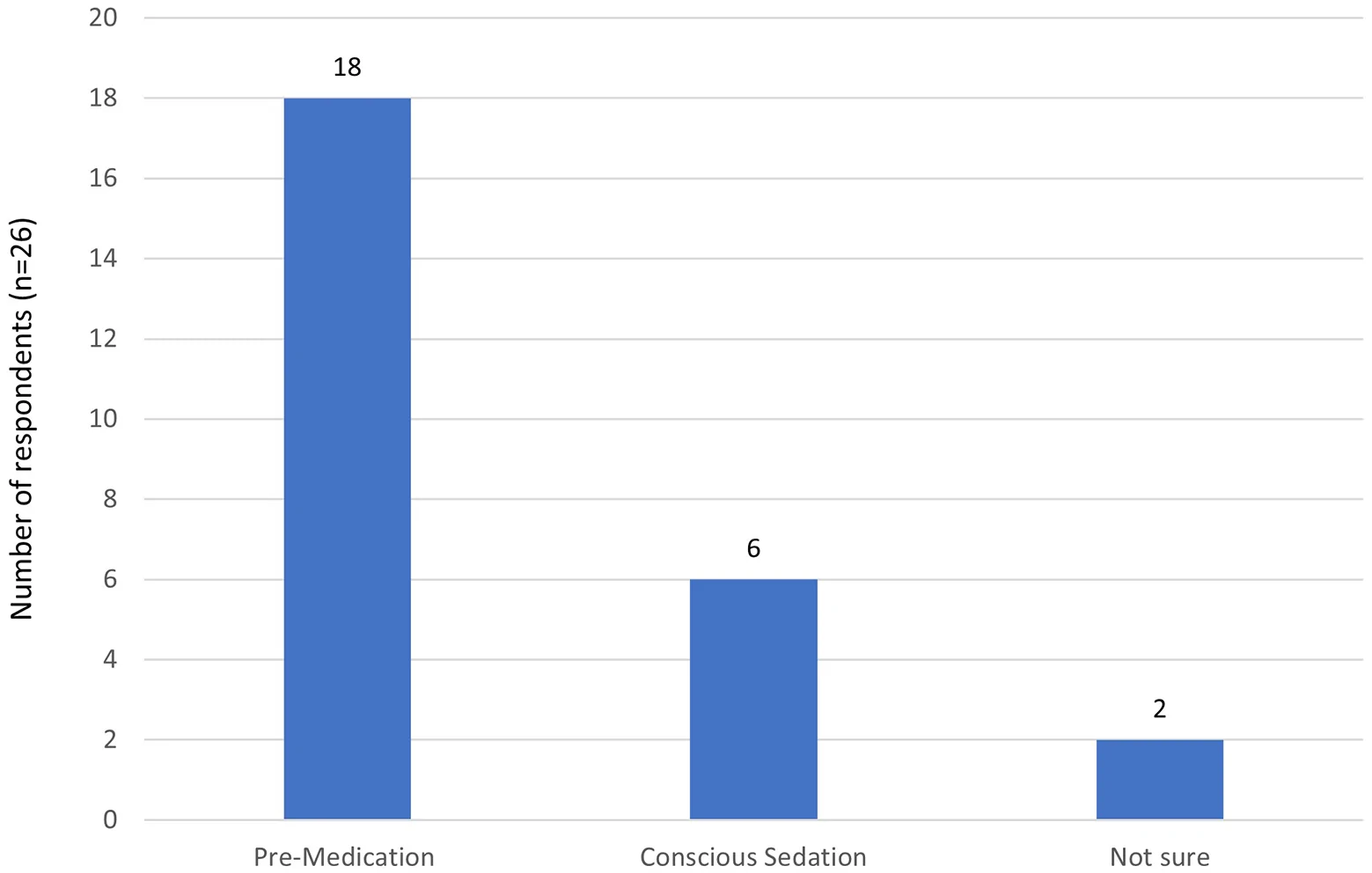
Patterns of OBZ prescribing.

**Figure 2.**
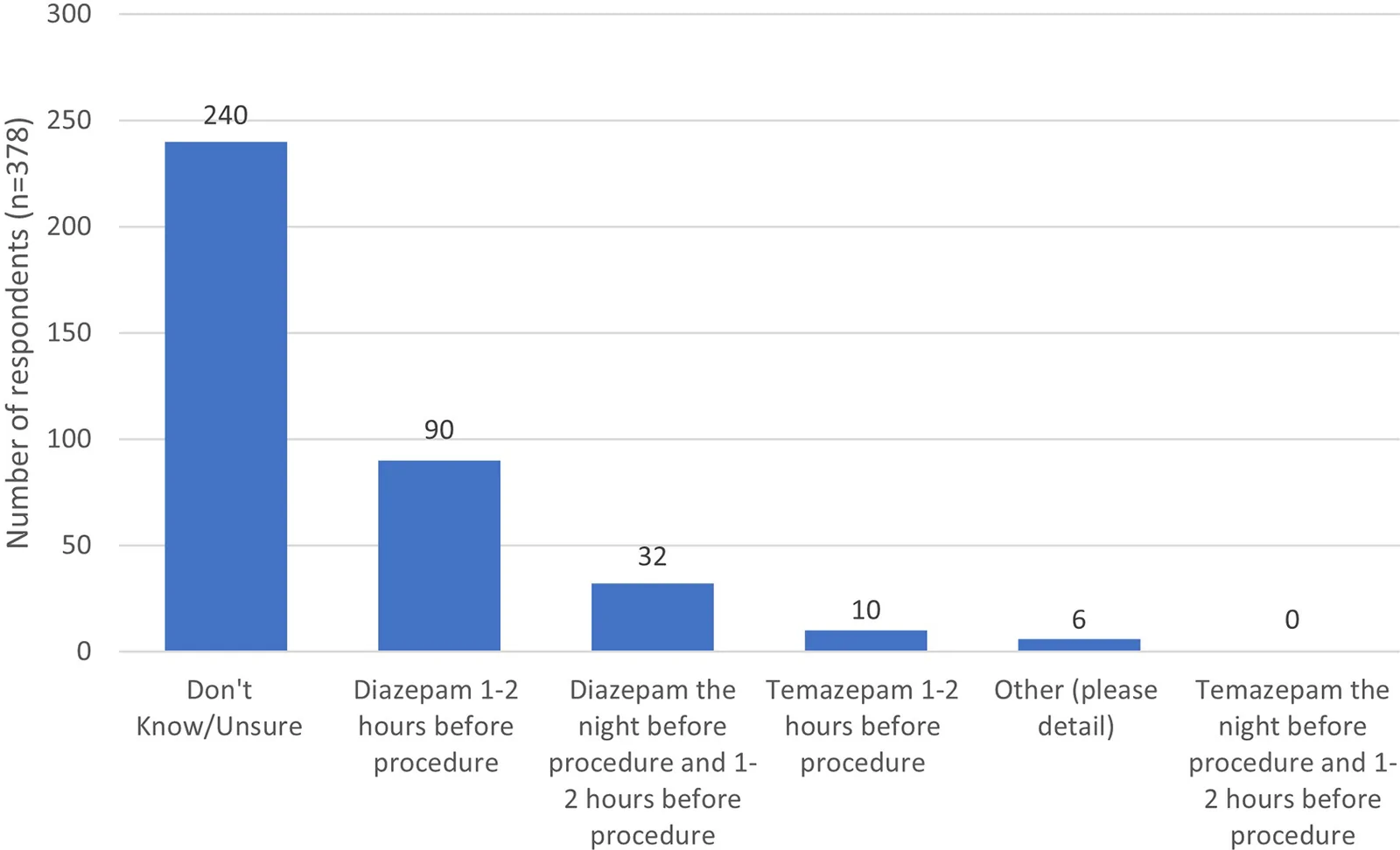
Preferred OBZ prescribing regimen.

Of 26 dentists who had prescribed OBZs, most prescribed as pre-medication (18/26 = 69.2%), six for conscious sedation, and two were not sure ( Figure 1). Just over two-thirds (n = 18) have done so in the last five years, while just under one-quarter (n = 6) reported that it was over five years ago. Over one-half of the dentists who did not prescribe them for anxiolysis (n = 55.6%, 210/378) would be interested in doing so in the future. Only 26 respondents (6.9%) reported having asked a general medical practitioner (GP) to prescribe OBZs as anxiolytics for a patient. In response to a scenario about OBZ prescribing for an anxiolytic, most didn’t know/were unsure (n = 240) ( Figure 2).

### 3.3 Barriers and enablers to OBZ prescribing

*Barrier: confidence in OBZ prescribing*

Most of the respondents (61.9%, n = 234/378) reported high or very high confidence in OBZ prescribing. The majority (77.8%, n = 294/378) wanted further training. It shows that even though they were confident, but still wanted training.

*Barrier: confusion about qualification requirements*

**Figure 3.**
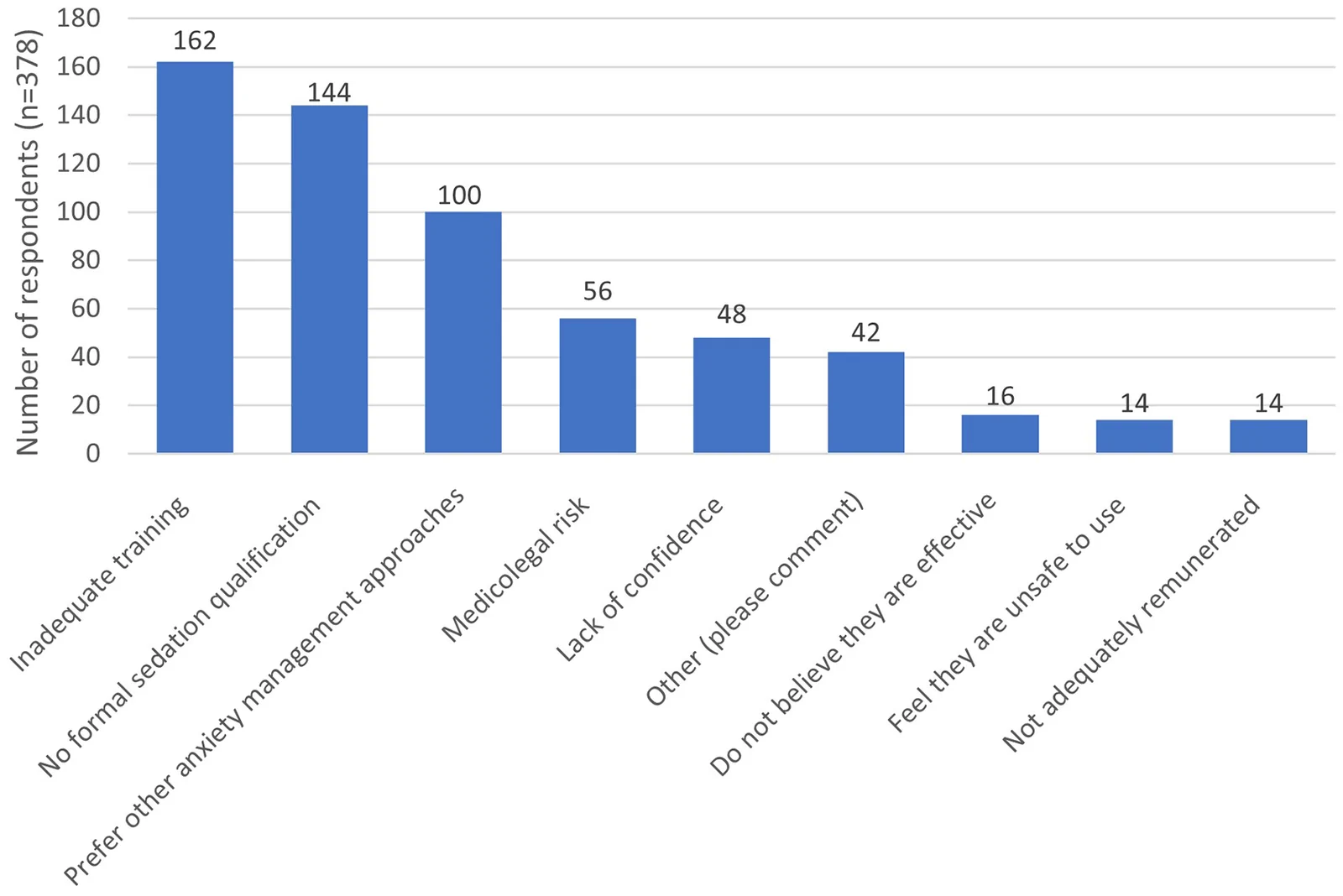
Barriers to prescribing OBZ.

Dentists who had never prescribed OBZs cited inadequate training (n = 162), not having a formal sedation qualification (n = 144), a preference for other anxiety management approaches (n = 100), medico-legal risk (litigation) (n = 56), and lack of confidence (n = 48) as top barriers to their prescribing ( Figure 3).

*Other factors: drug-seeking behavior*

**Figure 4.**
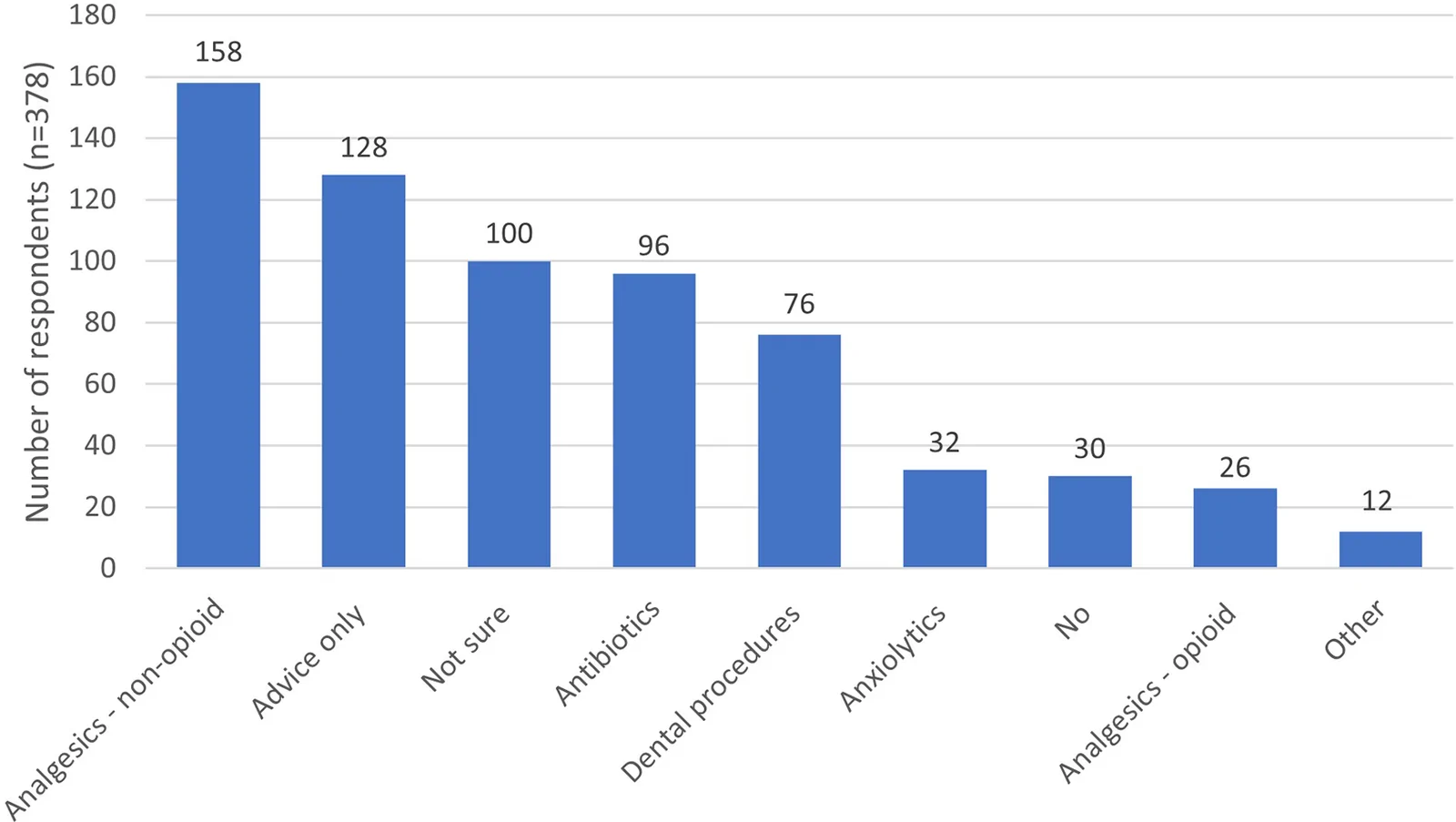
Other factors: drug-seeking behavior.

Anxiolytics were not commonly requested (n = 32). Respondents identified analgesics—non-opioid (e.g., paracetamol, ibuprofen)—as the most likely drugs to be requested by anxious dental patients (n = 158) and antibiotics (n = 96) ( Figure 4).

*Alternative approaches to anxiety management*

**Figure 5.**
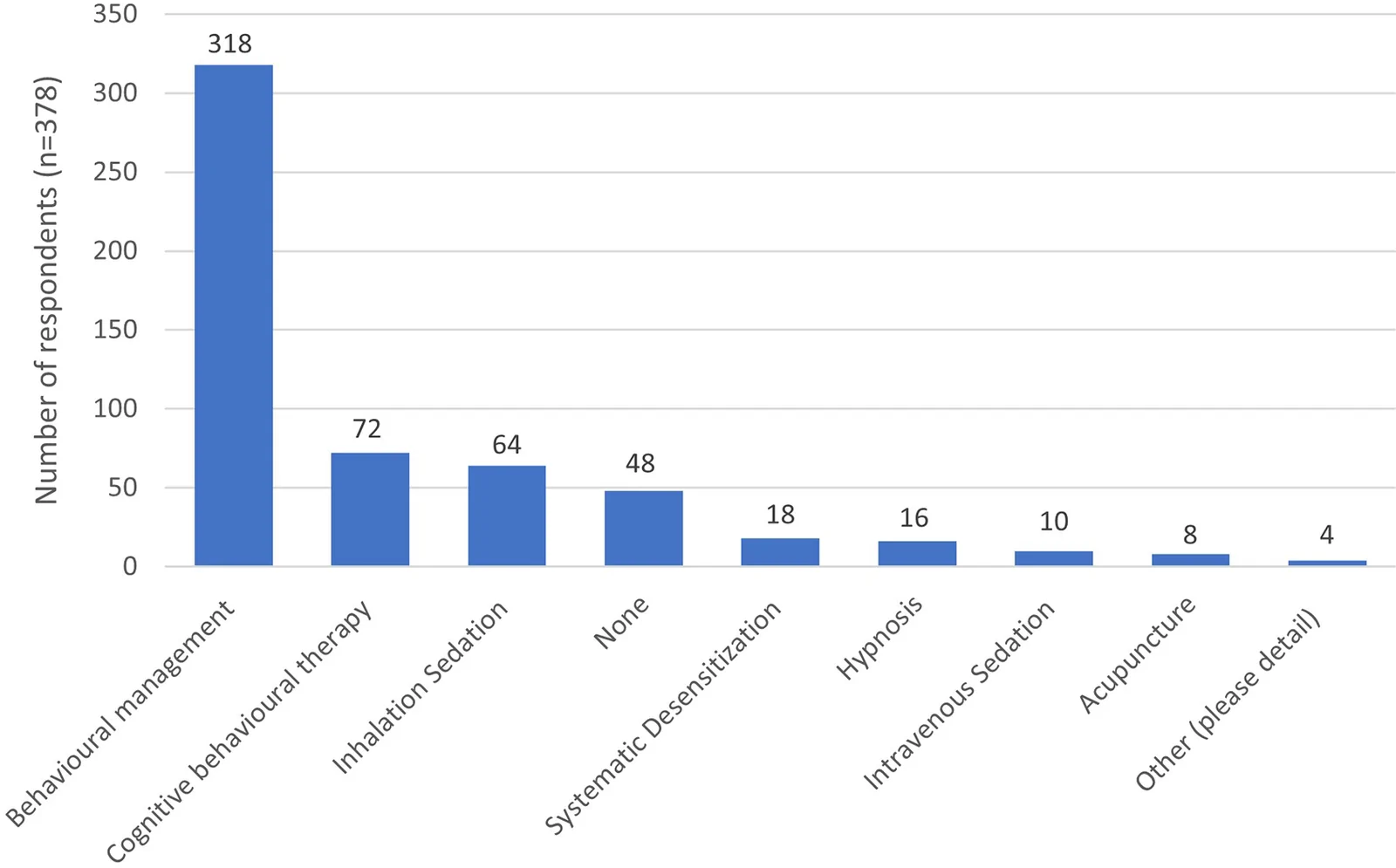
Alternative approaches to anxiety management.

Many dentists reported avoiding the use of OBZs, with most preferring behavioral management techniques ( Figure 5). A statistically significant association was found between barriers to prescribing OBZ (either as an oral pre-med or conscious sedation) for managing anxious patients and the type of dentist (p < .05). General dentists reported inadequate training, no formal sedation qualification, lack of confidence, do not believe they are effective, and not adequately remunerated as barriers to prescribing OBZ (either as an oral pre-med or conscious sedation) for managing anxious patients. On the other hand, specialty dentists/trainees reported that they prefer other anxiety management approaches, due to medicolegal risk, and felt they are unsafe to use as barriers to prescribing OBZ (either as an oral pre-med or conscious sedation) for managing anxious patients.

## 4. Discussion

This is the first study in the Kingdom of Saudi Arabia (KSA) to assess the prescription pattern of oral sedatives in dental practice. The primary aim of the study was to determine the prevalence of oral sedatives used for dental patients in the Eastern region of Saudi Arabia. Our result showed that the prevalence of OBZ prescription is 6.9%. This rate is considerably lower than the few studies reported earlier ( Table 4). Finn et al reported more than 50% prescribed OBZ among 235 dentists whom they have surveyed, where more than 90 % of dentists were general dental practitioners. ^10^ Teoh et al also conducted a study comparing the pattern of prescription in 3 countries between 2013-2018, with the USA considered to be prescribing more commonly than England and Australia. The US dental practitioners prescribed benzodiazepines 23 times more frequently, compared to English dentists, and 7 times more than Australian dentists. ^7^ Although this study does not provide direct prevalence, it does explain the commonality of prescribing OBZ. However, in another study conducted in France, which was not a direct prevalence study, they found that 9.2% of children received OBZ. ^15^ Another study done in Australia found an increase in the prescription rate of OBZ by 16% from the year 2013 to 2016. ^8^ However, a study done in Brazil showed a very low prescription rate, and the most frequently dispensed drugs were Bromazepam, Alprazolam, and Diazepam. ^11^

**Table 4.** Summary of the main findings of previous studies.

Author (year)	Country	Prevalence of prescriptions	Prescriptions/1000 population	Per capita rates (100,000 inhabitants)	The most common drug prescribed
Finn et al. (2022)	United Kingdom	50 %	0.11	-	Diazepam
Pouliquen et al. (2021)	France	9.2 %	-	-	Hydroxyzine
Teoh et al. (2021)	United states	-	3.10	-	Diazepam
Teoh et al. (2019)	Australia	-	0.50	-	Diazepam
Lino et al. (2010)	Brazil	-	-	6,832	Bromazepam

The low prescription prevalence in our study could be due to a general lack of training and formal qualifications, which was visible in the further answering of questions at the last part of the survey. Most respondents in our study reported high confidence in prescribing OBZ, but they wish to have further training. Further, many dental practitioners preferred other anxiety management approaches. Many practitioners requested other drugs, such as non-opioid analgesics and antibiotics, rather than anxiolytics. In addition, concern about the medicolegal risk appears to be a barrier that can be correlated to the low prescription rate of OBZ. A similar observation was seen in the study of Finn et al, where they have reported Inadequate training, confusion about guidelines, medico-legal risk, and issues of general practitioners prescribing anxiolytics to dental patients, unknown to their dentist, are the main barriers in prescribing OBZ.

The difference in the rate of prescription in different countries could also be due to differences in the guidelines of prescriptions of these medications and their experience gained from other countries. ^10^ One of our study findings showed that practitioners who are non-Saudi qualified (foreign trained) were more likely to prescribe OBZ. This shows the lack of exposure to such clinical practice among KSA-trained dentists. Also, maybe due to the strict regulations from the Saudi Ministry of Health (MOH) and Food and Drug Administration (FDA) regarding the prescription of anxiolytics, which can affect the prescription rate of this type of medication.

Intercollegiate Advisory Committee for Sedation in Dentistry (IACSD, UK) guidelines clearly state that conscious sedation can only be provided by dental practitioners who have received additional training. However, all dental practitioners can prescribe OBZs at anxiolytic doses as pre-medication. ^17^ American Psychiatric Association (APA) recommendations and guidelines for anxiety emphasize short-term use, the lowest effective dose, careful patient selection, and avoidance of long-term prescriptions whenever possible.

Practitioners who prescribed OBZ preferred to prescribe them pre-operatively. This factor appears to be similar to the previous reports of the pattern of prescription of OBZ. In the previously mentioned study in England, a similar observation was reported. ^10^ A few other studies which were regarding the OBZ prescription directed the use pre-operatively. ^12– 14^ Diazepam was the most prescribed drug out of OBZ, and more than 50% of their prescriptions were single doses 1 hour before the procedure as Pre-medication, which agrees with our study findings. ^8^ There was a small difference in prescribing between males and females. Older and more experienced practitioners were more likely to prescribe OBZ than younger and less experienced. This could be due to the introduction of new behavioral and cognitive management approaches, which can reduce the need for medication prescription. Also, a specialist/specialty trainee was more likely to prescribe OBZ. ^18^

We found a statistically significant association between barriers in prescribing OBZ and the type of dentist. In which general dentists expressed the lack of training, confidence, and formal qualification compared to specialists. Because of this, they preferred other management approaches. Their concern regarding the medicolegal risk was also considered to be a barrier. Similar findings were observed in the UK, where over 70 % of dentists expressed the need for extra training. In addition to this, they are also concerned about the lack of clear guidelines. Like our study findings, medico-legal risk, a preference for other anxiety management approaches, concerns about safety, inadequate remuneration, and concerns about drug effectiveness were also seen as barriers for prescription in their study. ^10^ Further interesting findings in our study are that referral of patients to general medical practitioners or physicians for the prescription of OBZ and/or management of anxiety. Although it appears to be legally safe, it reflects the lack of exposure to OBZ prescription among general dental practitioners. It also exerts additional workload on general medical practitioners. However, it may be that dental practitioners are not aware of the guidelines.

Our study is the first to address this topic in the Kingdom of Saudi Arabia, it is clear that a general lack of confidence about OBZ prescribing was seen, which added desire for further training in prescribing anxiolytics, even among the participants who answered having high or very high confidence in OBZ prescription. Further, the lack of training and unawareness of the confusion about the policy need to be addressed. Guidelines need to be clear without any ambiguity, which increases the confidence among general dental practitioners. ^10^ Furthermore, in the Kingdom of Saudi Arabia the general dentists are not privileged to prescribe benzodiazepines.

The major strength of this study is the first study to be reported from KSA, thus, it provides firsthand information regarding the prescription pattern of OBZ, which can be utilized by the policymakers and authorities to address the issue. The benefit of which is going to be transferred to the quality of life for dental patients. The limitation of the study is the mode of recruitment of participants, which was through social media. Although social media for recruitment allowed the survey to be conducted on a regional scale. In addition, participants could select more than one option, therefore, the total number of responses is greater than the number of respondents. Also, this was a survey-based study, which is confined to the Eastern region, and not representative of the entire region of Saudi Arabia. Our study is helpful and significant in identifying the barriers to prescribing OBZ. This led to the need for further study regarding the prescription of OBZ. As our study is a pathfinder, future studies are needed to confirm our findings, and comparisons should be made with other regions of the Kingdom.

Tackling anxiety, fear, and stress are daily concerns for dentists, since fear is the main barrier for seeking dental treatment. ^19^ Thus, it is essential to consider anxiety management and gaining confidence from the patient before treatment. Although we have seen a low prescription rate of OBZ compared to other countries, it does not mean that OBZ needs to be prescribed without considering the regulations. It has been seen that OBZ prescription has been substantially increased in the USA, along with this, complications, including the mortality rate related to these drugs, too. ^20^

The practitioners who do not prescribe OBZ follow different tools to limit and contain this stress, including positive communication, hypnosis, conscious parenteral and/or inhalation or orally administered sedation, and finally general anesthesia. ^21^ Therefore, a need does exist for the use of OBZ in dentistry that spans the international atmosphere. There is a segment of the patient population with fear and anxiety of dental treatment who may need this medication, and its appropriate use among dental practitioners needs to be increased with proper guidance and training.

## 5. Conclusion

Within the limitations of the study, it has been seen that the prevalence of prescribing OBZ in Saudi Arabia (Eastern Region) is low. The most important barriers identified by the practitioners were the lack of training and formal qualifications. There is a need for further attention to be devoted by health care specialty authorities and other related governing agencies to provide the required skills, knowledge, and training and authorization in the use of OBZ to improve the quality of life of dental patients in need of anxiety management before dental treatment.

## Ethical considerations

This study was approved by the Institutional Review Board (IRB) at Imam Abdulrahman Bin Faisal University with the letter number **IRB-PGS-2024-02-250** on 23 April, 2024.

## Informed consent statement

Written and Informed consent was obtained from all participants involved in the study.

## Data Availability

Figshare: Prevalence of benzodiazepine prescriptions in the dental office across the Eastern region of Saudi Arabia, https://doi.org/10.6084/m9.figshare.28945469. ^22^
- data.xlsx Figshare: Prevalence of benzodiazepine prescriptions in the dental office across the Eastern region of Saudi Arabia, https://doi.org/10.6084/m9.figshare.28945469. ^22^
- Appendix.pdf (contains the survey questionnaire) **License information:** CC BY 4.0
