# Supplementary material for: Prevalence of benzodiazepines prescription in the dental office across the Eastern region of Saudi Arabia
Source: F1000Res. 2025 Jul 25;14:732. [Version 1] doi: 10.12688/f1000research.165185.1 (PMC13557332; doi:10.12688/f1000research.165185.1)
Supplement: Peer review history

# Reviewer report: Reviewer response for version 1

**DOI:** 10.5256/f1000research.181788.r423826

**Published:** 2025-12-04

**Authors:** Ateet Kakti

**Affiliations:** 1 Riyadh Elm University, Riyadh, Saudi Arabia

Thank you for the opportunity to review this interesting and much-needed study addressing benzodiazepine prescription patterns among dentists in the Eastern region of Saudi Arabia. The topic is clinically relevant, especially considering the high burden of dental anxiety and the limited regional data. The manuscript is generally well structured, and the results are clearly presented.

 I do recommend full approval; however, the paper would benefit from several clarifications and minor revisions to strengthen readability, methodological transparency, and interpretation.

**1. Language and Clarity**

- Several sentences require grammatical correction or smoother phrasing to improve clarity.
  Examples include:
  - *“even though they were confident, but still wanted training*
  - Some sections of the discussion contain long sentences that can be simplified.

**2. Introduction **

- The introduction is comprehensive but could benefit from a clearer link between:
  - The prevalence of dental anxiety in KSA,
  - the need for sedation options, and
  - The gap in OBZ prescription patterns, specifically in the Eastern region.

**3. Methods**

- **Sampling**: Please clarify the exact recruitment method. Since the survey was distributed via social media and email, a brief statement about potential selection bias would strengthen transparency.
- **Questionnaire**: You mention it was adapted from a previous tool and validated. Please add 1–2 lines explaining which domains were validated and whether any modifications were made.
- **Language of the survey**: Since the survey was only in English, this could have limited participation among some practitioners. Adding this to the limitations would be appropriate.

**4. Results – Additional Explanation of Figures**

- Figures 1–5 are useful but require more explanation in the narrative text.
  For example:
  - **Figure 3**: The barriers displayed should be briefly summarized in the Results section to help readers interpret key differences.
  - **Figure 1**: Expand slightly on the distribution between pre-medication vs. conscious sedation.

**5. Statistical Analysis – Minor Clarifications**

- You mention chi-square and Fisher’s exact tests. Please specify which comparisons used Fisher’s test due to small cell sizes.

**6. Discussion Areas for Strengthening**

- The discussion is informative but could benefit from:
  - A clearer explanation of why foreign-trained dentists had higher prescribing rates (possibly greater exposure to sedation guidelines abroad).
  - An expanded discussion on the medicolegal concerns and training barriers, as these are central findings.

**7. Limitations Expand Slightly**

 The limitations section is mentioned, but could be strengthened. For example:

- Reliance on self-reported data may introduce recall or social desirability bias.
- An English-only questionnaire may have led to the exclusion of some practitioners.
- Recruiting through social media may not yield a fully representative sample.

Adding 2–3 lines addressing these would improve the credibility and transparency of the work.

**8. Conclusion – Minor Refinement**

- The conclusion is appropriate, but you may slightly expand on how policymakers or training institutions could respond to the identified gaps.

**Overall Recommendation:**

 This is a valuable and timely contribution, and with minor revisions to language clarity, figure interpretation, and expanded discussion of limitations, the manuscript will be even stronger. I commend the authors for addressing an understudied area in the region.

Is the work clearly and accurately presented and does it cite the current literature?

Yes

If applicable, is the statistical analysis and its interpretation appropriate?

Yes

Are all the source data underlying the results available to ensure full reproducibility?

Yes

Is the study design appropriate and is the work technically sound?

Yes

Are the conclusions drawn adequately supported by the results?

Yes

Are sufficient details of methods and analysis provided to allow replication by others?

Yes

Reviewer Expertise:

Dentistry

I confirm that I have read this submission and believe that I have an appropriate level of expertise to confirm that it is of an acceptable scientific standard.

# Reviewer report: Reviewer response for version 1

**DOI:** 10.5256/f1000research.181788.r411922

**Published:** 2025-09-15

**Authors:** Neeraj C. Deshpande

**Affiliations:** 1 Periodontology, Sumandeep Vidyapeeth Deemed to be University, Vadodara, Gujarat, India

1.⁠ ⁠Some grammatical errors and awkward phrasing reduce readability (e.g., “even though they were confident, but still wanted training”).

 2.⁠ ⁠Figures (e.g., barriers, prescribing patterns) are informative but under-explained in text.

 3.⁠ ⁠Limitations are acknowledged but understated. For instance, the reliance on English-only questionnaires may have excluded some practitioners.

 4. Article needs minor revision

Is the work clearly and accurately presented and does it cite the current literature?

Yes

If applicable, is the statistical analysis and its interpretation appropriate?

Yes

Are all the source data underlying the results available to ensure full reproducibility?

Yes

Is the study design appropriate and is the work technically sound?

Yes

Are the conclusions drawn adequately supported by the results?

Yes

Are sufficient details of methods and analysis provided to allow replication by others?

Yes

Reviewer Expertise:

Periodontology, Oral Implantology, Oral Health,

I confirm that I have read this submission and believe that I have an appropriate level of expertise to confirm that it is of an acceptable scientific standard.
